# Increased plasma apoM levels in the patients suffered from hepatocellular carcinoma and other chronic liver diseases

**DOI:** 10.1186/1476-511X-7-25

**Published:** 2008-07-24

**Authors:** Jingting Jiang, Xiaoying Zhang, Changping Wu, Xihu Qin, Guanghua Luo, Haifeng Deng, Minyang Lu, Bin Xu, Min Li, Mei Ji, Ning Xu

**Affiliations:** 1 Department of Tumor Biological Treatment, The Third Affiliated Hospital of Suzhou University, Changzhou 213003, PR China; 2 Department of Surgery, The Third Affiliated Hospital of Suzhou University, Changzhou 213003, PR China; 3 Comprehensive Laboratory, The Third Affiliated Hospital of Suzhou University, Changzhou 213003, PR China; 4 Section of Clinical Chemistry & Pharmacology, Lund University, S-221 85 Lund, Sweden

## Abstract

**Objective:**

To determine plasma apolipoprotein M (apoM) levels and other lipid profiles in hepatocellular carcinoma (HCC) patients compared to other chronic liver diseases and normal subjects.

**Materials and methods:**

36 HCC, 68 chronic hepatitis, 29 liver cirrhosis patients and 64 normal controls were subjected in the present study. Serum lipids, lipoproteins, apolipoprotein AI (apoAI) and apoB were determined by the conventional methods. Plasma apoM levels were semi-quantitatively determined by both dot-blotting and western blotting analysis.

**Results:**

Serum levels of triglycerides (TG), HDL-cholesterol, apoAI and lipoprotein (a) (Lp(a)) were significantly lower in the HCC patients than in the normal subjects, whereas there were no obvious differences on serum total cholesterol, LDL-cholesterol and apoB between HCC patients and normal subjects. However, plasma apoM levels in HCC patients were significantly increased than those in the normal subjects, but lower than those in the chronic hepatitis and cirrhosis patients.

**Conclusion:**

It is concluded that serum TG, apoAI, HDL-C and Lp(a) were significantly decreased in HCC patients than in controls, whereas plasma apoM levels were significantly increased in the HCC patients. Decreased serum TG, apoAI, HDL-C and Lp(a) may reflect the liver damage in HCC patients, whereas the clinical significance of increased plasma apoM levels in relation to HCC is not clear.

## Introduction

Hepatocellular carcinoma (HCC) is one of the most common fatal malignant tumors in China and in other Southeast Asian countries [1,2], which has been attributed to the high incidence of hepatitis B infection [3-5]. Most plasma apolipoproteins, endogenous lipids and lipoproteins are synthesized by liver, which depends on the integrity of cellular liver functions. Under normal physiological conditions, liver ensures homeostasis of lipid and lipoprotein metabolism. It has been demonstrated that plasma lipid profiles could be changed in the HCC patients [6]. In majority of the reports, plasma levels of triglycerides (TG), cholesterol, free fatty acids (FFA), high-density lipoprotein (HDL), low-density lipoproteins (LDL), lipoprotein (a) (Lp(a)), apolipoprotein AI (apoAI) and apoB were slightly to significantly decreased in the HCC patients, however, in certain cases plasma levels of TG and Lp(a) might even increase [7-9]. It has been suggested that analysis of plasma levels of lipids, lipoproteins and apolipoproteins in HCC patients may reflect the status of hepatic cellular impairments [8], and decreased serum levels of cholesterol and apoAI may indicate a poor prognosis [7-9].

As apoM is exclusively expressed in hepatocytes and kidney tubular cells [10,11], the plasma apoM levels may also be changed in the HCC patients and in other liver diseases. In the present study we examined plasma levels of lipids, lipoproteins, apoAI, apoB and apoM in the HCC patients compared with the other liver diseases and normal subjects.

## Materials and methods

### Patients and controls

36 surgical operated HCC patients (29 men and 7 women, aged from 29 to 83 years old, mean age is 57 years old), 68 chronic hepatitis patients (53 men and 15 women, 16–72 years old, mean age is 37 years old) and 29 liver cirrhosis patients (21 men and 8 women, 38–79 years old, mean age is 52 years old) were subjected in the present study. 64 normal subjects (44 men and 20 women, 28–71 years old, mean age is 43 years old) were selected as controls. All normal subjects were confirmed by blood biochemical tests, virus tests and B-type ultrasonic inspection to exclude hepatitis or other liver diseases. The present study was approved by the local ethics committee.

### Determinations of serum lipid profile and plasma apoM levels

Serum levels of TG, TC, HDL-cholesterol and LDL-cholesterol were determined by enzymatic method, and serum levels of apoAI and apoB were determined by the turbidity method. All samples were carried out on the Beckman LX-420 automatic biochemistry analyzer. Plasma apoM levels were semi-quantitatively determined by both dot-blotting and western blotting analysis with a specific rabbit anti-human apoM antibody [12]. In brief for dot blotting analysis, 2 μl plasma samples were applied to the Hybond-C membrane in triplicate. All samples were applied in the same membrane. The membrane was quenched in TBS buffer in the presence of 4% Tween and 3% BSA for 4 hrs, and then incubated with 1:4000 diluted primary antibodies at 4°C overnight. After washing 4 times with TBS buffer, it was incubated with AP conjugated goat anti-rabbit IgG antibody (Southern Biotech, USA) for 4 hrs at room temperature, and then visualized by NBT/BCIP method (Sino-American Biotechnology Co. Shanghai, China), according to the manufacturer's instruction. Plasma apoM levels were also determined by the western blotting analysis. 5 μl plasma was fractionated by SDS-polyacrylamide gel electrophoresis (SDS-PAGE), transferred to nitrocellulose membrane, and incubated with rabbit against human apoM polyclonal antibodies. AP conjugated goat anti-rabbit IgG antibody was used as the secondary antibody. The relative amount of apoM was analyzed by a scanner using Quantity One (Version4.2.1, Bio-Rad Laboratories, Hercules, USA) and represented as volume (intensity/mm^2^).

### Statistical analysis

Statistical analysis was performed with SPSS13.0 software. Differences of serum lipid profiles and plasma apoM levels between patients and controls were analyzed by the one-way ANOVA followed the Newman-Keuls multiple comparison tests. Data are expressed as means ± SE. A *p *value less than 0.05 (*P *< 0.05) was considered as significant.

## Results

### Serum lipid profiles in different liver diseases and in controls

As shown in table 1, both serum levels of Lp (a) and apoAI were significantly decreased in the patients suffered from HCC, chronic hepatitis or liver cirrhosis. And in HCC patients, serum levels of triglycerides and HDL-C were also statistically significantly decreased compared with those in normal subjects. There were no obvious changes on serum cholesterol, LDL-cholesterol and apoB between HCC patients and normal subjects. However, in the patients with liver cirrhosis, serum levels of TG and HDL-cholesterol were even higher than those in the controls.

**Table 1:** Lipid profiles in normal subjects and in the patients suffered from hepatocellular carcinoma (HCC), chronic hepatitis or liver cirrhosis

**Parameter**	**Means ± SD**	**F value**	**P value**	**vs. Normal**	**vs. HCC**	**vs. CH**
**T-Chol (mmol/L)**		4.761	0.003			
Normal (n = 64)	4.65 ± 0.87					
HCC (n = 36)	4.41 ± 1.85			0.439		
Chronic hepatitis (n = 68)	4.63 ± 1.00			0.929	0.480	
Liver cirrhosis (n = 29)	5.70 ± 2.62			0.002	0.001	0.001
						
**TG (mmol/L)**		19.337	0.000			
Normal	1.59 ± 0.81					
HCC	1.12 ± 0.57			0.001		
Chronic hepatitis	2.15 ± 0.64			0.000	0.000	
Liver cirrhosis	1.90 ± 0.64			0.044	0.000	0.101
						
**HDL-C (mmol/L)**		6.139	0.001			
Normal	1.30 ± 0.36					
HCC	1.06 ± 0.45			0.043		
Chronic hepatitis	1.27 ± 0.55			0.816	0.064	
Liver cirrhosis	1.66 ± 0.99			0.005	0.000	0.003
						
**LDL-C (mmol/L)**		0.896	0.444			
Normal	2.73 ± 0.66					
HCC	2.37 ± 0.79					
Chronic hepatitis	3.48 ± 6.04					
Liver cirrhosis	2.73 ± 1.08					
						
**Lp(a) (mg/L)**		7.770	0.000			
Normal	159.41 ± 123.06					
HCC	87.64 ± 56.39			0.000		
Chronic hepatitis	89.80 ± 54.75			0.000	0.910	
Liver cirrhosis	101.97 ± 119.23			0.006	0.537	0.555
						
**ApoAI (g/L)**		7.200	0.000			
Normal	1.24 ± 0.16					
HCC	1.13 ± 0.24			0.021		
Chronic hepatitis	1.08 ± 0.26			0.000	0.352	
Liver cirrhosis	1.03 ± 0.31			0.000	0.124	0.384
						
**ApoB (g/L)**		1.669	0.175			
Normal	1.07 ± 0.37					
HCC	0.90 ± 0.48					
Chronic hepatitis	1.02 ± 0.28					
Liver cirrhosis	1.07 ± 0.53					
						
**ApoM (Int/mm^2^)**		80.657	0.000			
Normal	433.70 ± 79.53					
HCC	712.87 ± 345.98			0.017		
Chronic hepatitis	1318.77 ± 752.34			0.000	0.000	
Liver cirrhosis	2252.46 ± 790.45			0.000	0.000	0.000

### Plasma apoM levels in HCC, chronic hepatitis, liver cirrhosis and normal subjects

**Figure 1:**
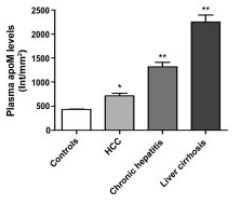
**Comparisons of plasma apoM levels in HCC patients, chronic hepatitis, liver cirrhosis and in controls.** Plasma apoM levels were determined by dot blotting analysis. Data are represented as means ± SE and it expressed as the intensity/mm^2 ^that was analyzed by the software of Quantity One. * < 0.05; ** < 0.01 vs. controls.

As shown in Fig 1, the plasma relative apoM levels in HCC patients were 712.87 ± 345.98 Int/mm^2^, which was significantly higher than those in the normal subjects (433.70 ± 79.53 Int/mm^2^) (t = 3.399, *P *< 0.05). ApoM levels were even higher in the patients suffered from chronic hepatitis and cirrhosis, the later with highest plasma apoM levels. In the present study plasma apoM levels were semi-quantified by both dot-blotting and western blotting analysis, and similar results were obtained from both methods.

## Discussion

In the present study it is demonstrated that both apoAI and Lp(a) were significantly decreased in the patients suffered from either HCC, chronic hepatitis or liver cirrhosis, which indicates that apoAI and Lp(a) can be considered as a stable index of liver damage. Interestingly in the present study, we demonstrated that plasma apoM levels were significantly increased in the HCC patients, whereas serum TG, apoAI, HDL-C and Lp(a) were significantly lower in HCC patients than in controls. Decreased serum TG, apoAI, HDL-C and Lp(a) may reflect the liver damage in HCC patients, however, the clinical significance of increased plasma apoM levels in relation to HCC is not clear.

The patients with HCC frequently have other liver diseases such as chronic hepatitis and/or cirrhosis. All these conditions (hepatitis and cirrhosis of the liver) are often associated with plasma lipid and lipoprotein aberrations [13]. In the present study we demonstrated that serum TG was significantly decreased in HCC patients than in the normal subjects, which was similar to the reported data that plasma TG decreased by 20–30% in the patients with HCC [14]. However, Alsabti, et al., [15] reported that serum TG in HCC patients were even increased when compared to those with cirrhosis. Ooi, et al., [8] reported that plasma TG levels in HCC patients were not significantly different compared with the controls. These results emphasize the fact that changes of plasma lipid profile may not always imply the presence of HCC and one need to exercise caution in interpreting these results.

About 80% endogenous cholesterol are synthesized in the hepatocellular microsomes that contain cholesterol synthesis enzymes [16,17]. In HCC and chronic liver diseases the synthesis and metabolism of cholesterol are impaired. It leads to a decrease in plasma cholesterol levels [8,14,18,19]. In the present study we demonstrated that total cholesterol, apoB and HDL-cholesterol were decreased in HCC patients, and there were no obvious changes on serum LDL-cholesterol in HCC patients compared with controls. Ahaneku, et al., [19] analyzed HDL-fraction levels including HDL-C, HDL-phospholipids (HDL-PL) and the ratio of HDL-C/HDL-PL, in HCC patients and compared with the controls. They found that plasma HDL-C, HDL-PL and HDL-C/HDL-PL were significantly lower in HCC patients than those in the controls. Motta, et al., [14] studied 40 patients with HCC, and evaluated the LDL-C, HDL-C. In patients with HCC, LDL-C level was significantly lower than in the controls. Kanel, et al., [20] reported that patients with primary or metastatic liver cancer had strikingly decreased HDL-C. Ooi, et al., [8] suggested that HDL-C may be clinically useful to reflect the pathologic conditions, and can be used to evaluate the severity of liver diseases.

Liver represents the main site of Lp(a) synthesis [21-23]. Half-life of Lp(a) is about 3.3–3.9 days in human plasma [24], which is influenced in the early stage when liver function was impaired [21]. Lp(a) is synthesized and metabolized independently of other plasma lipoproteins, and Lp(a) level is not influenced by various dietary manipulations [25]. It has been reported that serum Lp(a) were significantly lower in the HCC patients [26,27]. The similar results were obtained in the present study. However, Geiss, et al., [28] observed patients with acute hepatitis showed a marked increase in Lp(a) concentration, i.e., 7 mg/dL in acute stage and 32 mg/dL in the convalescence of the disease. Basili, et al., reported that Lp(a) could also be increased in the patients suffered from HCC together with cirrhosis [29]. It has been demonstrated that Lp(a) together with ferritin and alpha-fetoprotein could be a sensitive and early marker to evaluate liver function [14]. As Lp(a) has positive correlation with the hepatic status, it has been suggested that Lp(a) could be considered as a index of liver function [14,23,30].

Liver is the main organ for the synthesis, storage, transportation and degradation of certain apolipoproteins [31]. Each protein may be influenced by liver disease in a different way, and serum lipoprotein concentrations with faster turn-over are more reduced with respect to those with slower turn-over [32]. It is reported that serum concentrations of apoAI and apoAII were significantly lower in the patients suffered from HCC [7,9], but an increase in the proportion of proapoAI was found in patients with HCC [33]. In the present study we demonstrated that both apoAI and apoB were significantly decreased in HCC patients compared with the normal subjects. Decreased serum apoAI and apoB levels may reflect the liver damage in HCC patients, as most HCC patients are companied with chronic liver injury. Interestingly in the present study we demonstrated that plasma apoM levels were significantly increased in the HCC patients than in controls, and apoM levels were even higher in the patients suffered from chronic hepatitis and liver cirrhosis, which may relate to the aberration of host immune system. The detailed mechanism needs further investigation.

## Competing interests

The authors declare that they have no competing interests.

## Authors' contributions

JJ designed the study and drafted the manuscript. XZ and CW participated in the experimental instruction. XQ provided clinical samples. GL, HD and ML carried out data analysis and figure formatting. BX, ML and MJ performed all of the experiments. NX drafted the manuscript, designed the study and coordination. All authors have read and approved final manuscript.
